# Combination of RNA Interference and Stem Cells for Treatment of Central Nervous System Diseases

**DOI:** 10.3390/genes8050135

**Published:** 2017-05-06

**Authors:** Xue-Qin Hou, Lei Wang, Fu-Gang Wang, Xiao-Min Zhao, Han-Ting Zhang

**Affiliations:** 1 Institute of Pharmacology, Taishan Medical University, Taian 271016, Shandong, China; houxueqin09@126.com (X.-Q.H.); wang.lei1118@163.com (L.W.); wfg713@sina.com (F.-G.W.); zhaoxiaominty@163.com (X.-M.Z.); 2 Departments of Behavioral Medicine & Psychiatry and Physiology & Pharmacology, Blanchette Rockefeller Neurosciences Institute, West Virginia University Health Sciences Center, Morgantown, WV 26506, USA

**Keywords:** RNA interference, miRNA pathway, stem cells, differentiation, central nervous system diseases

## Abstract

RNA interference (RNAi), including microRNAs, is an important player in the mediation of differentiation and migration of stem cells via target genes. It is used as a potential strategy for gene therapy for central nervous system (CNS) diseases. Stem cells are considered vectors of RNAi due to their capacity to deliver RNAi to other cells. In this review, we discuss the recent advances in studies of RNAi pathways in controlling neuronal differentiation and migration of stem cells. We also highlight the utilization of a combination of RNAi and stem cells in treatment of CNS diseases.

## 1. Introduction

Central nervous system (CNS) diseases such as Alzheimer’s disease (AD), Huntington’s disease (HD), and spinal cord injury (SCI) are characterized by neuronal loss in the brain or spinal cord, which cause corresponding impairments in neural functions [1,2,3]. The discovery of stem cells incited expectation for the treatment of these diseases. Stem cells have the ability to renew themselves and differentiate into neurons [4,5] with no immunogenicity [6]. Moreover, stem cells have the capacity of migration towards the sites of injury [7,8,9]. Thus, neuronal loss could be remedied to a certain extent when stem cell therapy is employed, and cell-based therapy is becoming increasingly attractive in the study of CNS diseases. However, stem cells could differentiate into other cell types (such as astrocytes and oligodendrocytes) besides neurons [10]. This disadvantage could limit the application of stem cells in treatment of CNS diseases. Therefore, controlling lineage-specific genes in stem cells has been considered to be a strategy to direct stem cell differentiation. Conventionally, the strategy is regulating the lineage-specific inductive factors [11,12,13,14]. However, the signaling cascades are complex since they include not only inductive factors, but also repressive regulators. Thus, it is possible that controlling the negative genes in stem cells could promote the differentiation of specific lineage commitment [15]. RNA interference (RNAi) is considered an important approach for controlling gene expression [16], as well as for gene therapy [17]. However, the safety and efficiency of RNAi therapy still needs to be considered. The interaction between stem cells and other cells could lead to a more effective delivery of RNAi from stem cells to target tissues [18]. Therefore, the combination of RNAi and stem cells has therapeutic potential for neurological diseases. This brief review focuses on the RNAi pathways in governing the behavior of stem cells by targeting lineage-specific genes. We will also discuss the combination of RNAi and stem cells as a potential therapeutic approach in CNS diseases.

## 2. Endogenous RNAi Pathways in Governing the Neuronal Differentiation and Migration of Stem Cells

RNAi is a mechanism to inhibit gene expression by double-strand RNA (dsRNA) [19]. Gene-silencing can be mediated by small RNAs, including small interfering RNA (siRNA), short hairpin RNA (shRNA), and microRNA (miRNA) [20,21]. RNAi has been used as a possible therapeutic strategy, and efforts have been made to translate RNAi into clinical applications [20,22,23,24,25]. Additionally, recent studies have identified some miRNAs and their target genes as crucial players in self-renewal and differentiation of stem cells. Therefore, understanding how RNAi pathways are mediated by miRNAs in stem cells may help in discovering more target genes.

Stem cells, such as mesenchymal stem cells (MSCs) and neural stem cells (NSCs), are a potentially promising source for cell-based therapy. MSCs can be easily derived from bone marrow [26], umbilical cord [27], and adipose [28]; they can differentiate into various cells, such as neurons [29], chondrocytes [30], and adipocytes [31]. Bone marrow-derived MSCs (BMSCs) were reported to downregulate miR-124, one of the neuron-specific miRNAs abundantly expressed in the brain [32]. On the other hand, upregulation of miR-124 in BMSCs increases expression of neuronal markers, including β-III tubulin, MAP-2, and synaptophysin, and the rate of differentiation of the transplanted BMSCs into neurons in the injured site of spinal cord in rats [33]. Interestingly, miR-124 was regulated by the transcriptional repressor REST (RE1-silencing transcription factor) [34], which could prevent precocious neuronal differentiation and maintain neural stem cells (NSCs) self-renewal in cultured adult NSCs [35]. These studies suggest that the REST-miR-124 pathway may play an important role in mediating neuronal production. Moreover, some of the miR-124 downstream targets have been reported, including Sox 9 [36], PTBP-1 [37], and small C-terminal domain phosphatase 1 (SCP-1) [38]. Sox 9 may determine the glial fate due to its capability of switching neural stem cells from neurogenesis to gliogenesis [39]. Delivery of exogenous miR-124 decreases expression of Sox 9 by targeting its 3′-UTR and increases neuronal differentiation of human neural progenitor cells (NPCs) [40]. In addition, it has been found that Sox 9 controls the induction of nuclear factor-I A (NFIA), which then forms a complex with Sox 9 to co-regulate a set of genes induced following glial initiation [41]. NFIA is also essential for inhibition of neurogenesis and facilitation of glial-fate specification in embryonic spinal cord progenitors [42]. Most recently, it has been found that NFIA is a target of miR-153, inhibition of which in early neurogenic neural stem/progenitor cells induces precocious gliogenesis [43]. In addition, NFIA is also a target of miR-223, which suppresses the proliferation of glial precursors via repression of NFIA in the CNS development [44]; the miR-223/NFIA axis inhibits tumorigenesis in a human glioma cell line. Further, overexpression of miR-223 reduces neuronal differentiation of human embryonic stem cells [45]. PTBP1, also known as PTB or hnRNP I, is considered a repressor of nervous system-specific splicing, and is downregulated by miR-124 during neuronal differentiation [37]. SCP-1, a member of SCPs family, is expressed in non-neuronal tissues. As neurons differentiate, REST/NRSF are reduced (neuron-restrictive silencer factor). While the presence of SCP-1 is not able to disrupt neuronal differentiation of P19 mouse embryonic stem cells, recruitment of SCP-1 by REST/NRSF regulates the neuronal gene silencing in non-neuronal cells [46].

Other miRNAs, such as miR-29a and miR-9, are also involved in enhancing the neural differentiation of BMSCs. Similar to miR-124, miR-29a also regulates neurogenic markers in BMSCs through targeting REST [47]. It is said that miR-29a upregulation results in decreases in REST [47]. Thus, neuronal differentiation is mediated through the miR-29a-REST pathway. MiR-9 promotes the neural differentiation of mouse BMSCs via targeting zinc finger protein 521 [48], which is enriched in most immature cells and reduced with differentiation [49].

There are many other miRNAs that modulate neuronal differentiation in various types of stem cells. miR-125b, one of the brain-enriched miRNAs, has been identified as a player in regulating neuronal differentiation [50,51]. In neural stem/progenitor cells (NS/PCs), miR-125b mediates proliferation, differentiation, and migration, through suppressing Nestin expression [52]. Additionally, miR-125b regulates synapse structure and function via targeting the NMDA receptor subunit NR2A, which is associated with a translational repressor fragile X mental retardation protein (FMRP) [53]. miR-20 promotes neural progenitor cell differentiation through inactivation of the REST-Wnt pathway [54], while overexpression of miR-128 reduces doublecortin levels in differentiating adult neural progenitors [55].

MiR-506-3p negatively regulates the expression of the transcription factor T-cell factor-3 (TCF3) [56], which was proved to be an integral component of the core regulatory circuitry of embryonic stem cells [57]. It has been shown that overexpression of miR-506-3p increases NSC proliferation and differentiation via targeting TCF3 [56]. In proliferating NPCs, the level of miR-19 is highly expressed, and decreased upon differentiation. Furthermore, miR-19 regulates cell migration by targeting Rap guanine nucleotide exchange factor 2 (Rapgef2) [58]. Overexpression of miR-7 promotes neural lineage determination and increases synapsin in human embryonic stem cell-derived neurons [59]. Recent studies also have identified some miRNAs that influence neuronal differentiation of human Wharton’s Jelly mesenchymal stem cells (WJ-MSCs), including miR-1290, miR-26b, miR-194, miR-124a, miR-4521, and miR-543; while the first four of which were upregulated, the last two were downregulated [60].

It has been demonstrated that different miRNAs could target the same gene and produce synergistic effects. For example, miR-124, -128, and -137 synergistically regulate the overlapping gene Sp1, whose level is decreased as cells differentiate. Silencing Sp1 reduces neuronal differentiation of NSCs and affects their viability and proliferation [61]. Taken together, the mechanism of differentiation and migration of stem cells is complex and involves various miRNAs and their target genes.

## 3. Combination of RNAi and Stem Cells for Treatment of CNS Diseases

RNAi is considered as an important approach to controlling gene expression [16]. However, one of the problems we confront is the enhancement of the safety and efficiency of RNAi therapy. Recently, combinations of gene therapy and stem cells have been considered to be a promising approach to the treatment of diseases [62]. Stem cells can be utilized as vectors for RNAi delivery as they have been shown to be able to deliver RNAi to other cells [18]. One of the critical barriers for stem cells though is to develop an easy, effective, and safe method to direct cell lineage-specific differentiation [16]. In order to do this, target genes are transfected into stem cells for overexpression. It should be noted that the RNAi signaling pathways play an important role in regulating differentiation, migration, and proliferation of stem cells. Therefore, a combination of RNAi and stem cells are of therapeutic potential for neurologic diseases.

### 3.1. Alzheimer’s Disease

Alzheimer’s disease (AD) is one of the most devastating, age-related neurodegenerative disorders [63]. The amyloid precursor protein (APP) and its product amyloid-β (Aβ) play central roles in AD pathogenesis. Additionally, the accumulation of APP products is associated with β-secretase (BACE1) activity. APP is cleaved by BACE1 and α-secretase to produce APP-C99 and APP-C83, and further produce Aβ and p3, respectively, by γ-secretase [64]. Therefore, reducing the cleavage of APP and the activity of BACE1 has great therapeutic potential for treatment of AD. It has been reported that decreased BACE1 levels using lentiviral vectors expressing BACE1 siRNA reduce Aβ production and AD-like alterations in APP transgenic mice [65]. Xie and co-workers [64] investigated the effects of APP adaptor proteins with phosphotyrosine-binding domains (ShcA, ShcC, and Fe65) expression on APP processing and Aβ production by using RNAi. Their study showed that the silencing of ShcC decreased the levels of APP-C-terminal fragments (APP-CTFs), Aβ, and BACE1 in H4 human neuroglioma cells stably overexpressing full-length APP (H4-FL-APP cells), while silencing of the homolog ShcA had no effect. RNAi silencing of Fe65 increased APP-CTF levels, while it also decreased Aβ levels in H4-FL-APP cells. These studies demonstrate that blocking APP processing, BACE1 activity, or Aβ production may provide therapeutic strategies against AD. Interestingly, the abnormally high levels of BACE1 protein expression appear negatively correlated with the expression of miR-29a/b-1/c cluster in AD [66,67]. Thus, miR-29 could be a potential target for intervention of AD. This is supported by a recent study [68] proving that recombinant pre-miR-29b using polyplexes decreases the BACE1 (around 80%) and Aβ42 (approximately 40–50%) expression levels in N2a695 cells, which is expected to improve the methodologies of miRNA-based therapeutics. In addition, it has been shown that downregulation of miR-98-5p decreases sorting nexin 6-dependent levels of Aβ in vitro, suggesting that miR-98-5p may be a therapeutic target for AD [69].

Neuronal loss is involved in the pathologies of AD and progressively promotes cognitive and memory impairment [1]. An in vitro study using MSCs to treat AD has shown enhancement of autophagy and clearance of amyloid-β (Aβ) in AD models [70]. Interestingly, transplantation of NSCs in APP/PS1 transgenic mice, an animal model of AD, does not alter amyloid pathology, but improves cognition [71]. When NSCs are genetically modified to express neprilysin (sNEP), the Aβ-degrading enzyme, amyloid pathology is reduced and the synaptic density is increased [72]. These results indicate that genetically-modified stem cells may hold great promise for the treatment of AD.

Since neuronal loss is an important feature of AD, one problem is figuring out how to induce neuronal differentiation of stem cells by genetic modification. As stated above, the transcription factor SOX9 is an important regulator in neuronal differentiation of stem cells. Knockdown of SOX9 in stem cells via nanotopography-mediated siRNA induces differentiation of NSCs into neurons more than into glial cells [16], leading to suppression of glial traits. This study demonstrated that SOX9 suppression in stem cells promotes neuronal differentiation. There are many other factors known to play a role in stem cell behaviors. As the principal active ingredient of vitamin A, retinoic acid (RA) exerts biological activities that promote neuronal differentiation of NSCs [73,74]. The RA-encapsulating poly (e-caprolactone) (PCL) nanofiber enhances mRNA expression of neural markers and contributes to MSC phenotypic changes towards the neural lineage [75]. Notably, the neuronal promoting effect of RA was found to be counteracted by the SOX9 protein in NSCs [76]. Therefore, inhibition of SOX9 expression may avoid the negative effect of SOX9 on RA. Recently, traceable PHEMA-RA-PCB-CPP/SPIONs/siSOX9 nanoparticles (ABC/SPIONs/siSOX9 NPs) have been developed for treatment of AD. The complex NPs control siSOX9 delivery, which is faster than RA, leading to downregulation SOX9 protein expression [77]. Therefore, ABC/SPIONs/siSOX9 NPs can efficiently control the differentiation of NSCs into neurons. Moreover, after transplantation of NSCs treated with ABC/SPIONs/siSOX9 NPs, APPswe/PS1dE9 transgenic AD mice display enhanced spatial learning and memory in the Morris water-maze test and increased neurons in the hippocampus as indicated by Nissl staining analysis [77]. The results from these studies suggest that SOX9 RNAi, together with RA, may enhance the neuronal differentiation of stem cells and replace degenerated neurons.

### 3.2. Huntington’s Disease

Huntington disease (HD) is a hereditary, progressive autosomal-dominant neurodegenerative disease caused by an exon 1 CAG trinucleotide-repeat-expansion mutation in the huntingtin (*htt*) gene [78,79]. HD is characterized by motor disturbance, cognitive dysfunction, and psychiatric features [80]. The pathological process is associated with neuronal loss [2]. *Htt* plays a crucial role in neuronal fate. It was reported that NSCs with expanded expression of CAG repeats causes neuronal deaths, while silencing the *htt* gene decreases neurons and increases astrocytes [81]. shRNA-mediated RNAi of mutant human *htt* in the animal model of HD improves behavioral and neuropathological abnormalities [82]. Additionally, nonallele-specific silencing of both mutant and wild-type *htt* via RNAi could improve motor coordination and survival in HD mice [83]. By examining the effects of post-symptomatic RNAi treatment in the HD model mice, it was found that silencing of the *htt* gene successfully ameliorated the neuropathological abnormalities (insoluble protein accumulation and downregulation of DARPP-32 expression) [84]. However, patients with HD might express both mutant and wild-type *htt* alleles. It seems necessary to allele-selectively inhibit mutant *htt* expression. Recently, another study demonstrated that [85] RNAi by single-stranded silencing RNAs (ss-siRNAs) potently (100-fold more than unmodified RNA) and allele-selectively (>30-fold) inhibited mutant *htt* expression in cells derived from HD patients; it also selectively reduced mutant *htt* allele throughout the brain in a mouse HD model. In addition, allele-selective silencing was induced by targeting the heterozygous single-nucleotide polymorphism (SNP) rs362331 in exon 50 and total *htt* silencing by miH12 both in vitro and in vivo [86]. To further clarify the extent of *htt* mRNA lowering in individual neurons, Keeler AM et al. [87] investigated the effect of miR^htt^ on *htt* mRNA levels in striatal neurons of Q140/Q140 knock-in mice, another HD model. They found that intrastriatal infusions of AAV9-GFP-miR^htt^ vectors reduced *htt* mRNA in striatum through a partial reduction in mutant *htt* mRNAs per cell in medium spiny neurons. Recently, miRNAs such as miR-10b-5p, miR-128a, and miR-34b-a have been proven to be associated with HD [88,89,90]. *Htt* gene expression is regulated by miRNAs and certain heterogeneous miRNA variants are functional and regulate the same target as canonical miRNAs [91]. Taken together, these studies demonstrate the feasibility of treating HD by using RNAi approaches. However, further problems are the poor uptake of RNAi and the transient effects when delivered systemically [92]. Stem cells can help solve these issues because they have been proven to deliver exogenous RNAi materials to other cells. It has been shown that fluorescent-labeled miR-124 and miR-145 mimics are efficiently delivered from MSCs to co-cultured NPCs and astrocytes [40]. To explore a cell-based platform for treating HD, a combination of RNAi and stem cells was employed in a recent investigation. The results showed that MSCs expressing shRNA antisense to *htt* transferred RNAi to the co-culture U87 cells (previously transduced with mutant *htt* fragment) and SH-SY5Y cells, leading to decreased levels of mutant *htt* expressed in the co-culture cells [18].

### 3.3. Spinal Cord Injury

Spinal cord injury (SCI) impacts a patient’s physical, psychological, and social well-being due to the traumatic event [93]. Approximately 1.7 million individuals worldwide suffer from SCI each year [94], with increases health care and living expenses [95]. It has been suggested that miRNAs regulate gene expression and are associated with the pathogenic processes of SCI, such as inflammation, oxidation, demyelination, and apoptosis [96]. Thus, miRNAs may become potential targets for the therapeutic intervention following SCI. Theis et al. [97] found that transfection of miR-133b into hippocampal neurons stimulated neurite outgrowth in vitro, and injections of lentivirus encoding miR-133b into the lesion site improved locomotor recovery after SCI in mice. Louw et al. [98] developed chitosan/miR-124 polyplex particles and showed that it could inhibit neuronal inflammation after microinjections into injured rat spinal cords.

Currently, standard therapies only have limited effects on secondary neuronal injury [94]. Thus, strategies for treatment and prevention of secondary neuronal damage are necessary. It has been known that neuronal loss is characteristic of SCI and that transplantation of stem cells affects proliferation and differentiation of endogenous stem and progenitor cells [3]. Stem cell-based therapy has been demonstrated to have therapeutic potential in SCI [99]. Given that miRNAs play an important role in the differentiation of stem cells [33], BMSCs were examined for the effect of miR-124 overexpression, which showed that transplantation of miR-124-transfected BMSCs into the injured rat spinal cord increased the number of neuronal cells and substantially improved the motor function of the hind limb of rats with SCI. These findings encourage targeting miRNAs for enhancing the therapeutic efficacy of stem cell transplantation for SCI. In addition, the proliferation, differentiation, and migration of stem cells are mediated by various factors and genes, including REST [35], Nogo receptors [100,101], and Leucine-rich repeat and immunoglobulin domain-containing protein (LINGO)-1 [102]. Thus, development of genetically engineered stem cells targeting these genes may enhance the therapeutic efficacy of stem cell-based therapy.

As mentioned previously, some miRNAs are involved in REST signaling pathways and play a negative role in regulating behavior of stem cells. Therefore, silencing of the REST gene increases expression of mesendoderm differentiation markers [103] and enhances neural stem/progenitor cell (NPC) neuronal commitment [15,104]. When the expression of REST is decreased, the transcription of TAC1, a neurotransmitter gene, is induced in human MSCs [105]. Moreover, the induction of TAC1 regulates neuronal differentiation of MSCs, and promotes neural regeneration and functional recovery in a model of SCI in zebrafish after transplantation with neural-induced MSCs [106].

Nogo-66 receptors (NgR) bind to three myelin-associated neurite outgrowth inhibitors (Nogo, myelin-associated glycoprotein, and oligodendrocyte myelin glycoprotein), causing inhibition of axon regeneration after injury in the adult mammalian CNS [107,108,109]. Deleting one or all of the three inhibitors does not enhance axon regeneration after spinal cord injury [110]. Interestingly, RNAi-mediated silence of the NgR gene in bone marrow mesenchymal stem cells before transplantation increases the growth of nerve fibers, improves morphology and behavioral performance, and reduces mortality in rats with spinal cord injury [111]. It has been demonstrated that p75 and LINGO-1 interact with NgR as co-receptors for the three myelin-associated neurite outgrowth inhibitors [112,113]. LINGO-1 is a nervous system-specific transmembrane protein expressed in neurons with high levels in the brain and low levels in the spinal cord, but is not detectable in non-neural tissues [113]. Additionally, recent studies have shown that LINGO-1 is expressed in NSCs, and its neutralization regulates NSCs maturation to neurons [114]. Moreover, inhibition of LINGO-1 via shRNAs increases differentiation of NSCs into neurons [115] and improves functional recovery and nerve regeneration in SCI rats [116]. These results indicate that inhibition of LINGO-1 is a potential treatment approach for SCI. Most recently, it has been shown that transplantation of LINGO-1-RNAi-treated NSCs increases tissue repair and functional recovery in rats subjected to SCI [102].

The high proportion of cell apoptosis is one of the main problems in cell transplantation. P75, a neurotrophin receptor, has been proven to induce apoptosis of neurons [117]. P75-suppressed BMSC decreases the level of apoptosis during neural differentiation in vitro [118]. Furthermore, transplanting p75 siRNA transfected BMSC in SCI rats could promote functional recovery [119]. These studies provide a clear indication that specific genes modified by RNAi can regulate differentiation of stem cells into neurons and increase the survival rate after neural differentiation; genetically modified stem cells can be a source of cells for treatment of SCI.

In addition to neuronal loss, oligodendrocyte cell death and demyelination are also important pathological characteristics of SCI [120]. Oligodendroglial precursor cells (OPCs) are considered the main source for differentiating into mature myelinating oligodendrocytes [121]. Thus, promoting differentiation and remyelination of OPCs could be a target for repair after SCI. TROY, a member of the tumor necrosis factor receptor superfamily (TNFRSF), is broadly expressed in postnatal and adult neurons. TROY can replace p75 in the p75/NgR1/LINGO-1 complex to activate RhoA in the presence of myelin inhibitors [122]. Notably, myelin mediates inhibition of OPC differentiation and impairs CNS remyelination [123,124]. It has been shown recently that TROY is expressed in the oligodendrocyte lineage on various differentiation stages, and negatively regulates OPC differentiation and myelination [125]. Transplantation of OPCs treated with TROY-RNAi for 72 h in rats with SCI significantly improves the therapeutic efficacy, resulting in evident morphological and neurological recovery.

## 4. Conclusions

**Figure 1:**
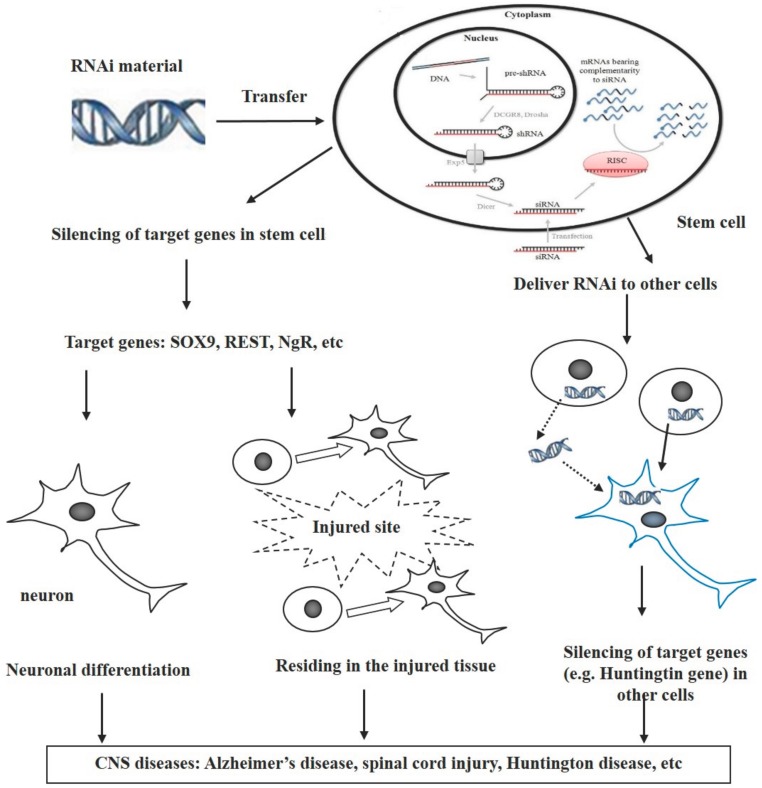
Use of stem cells as vectors for transfer of RNAi in treatment of CNS diseases. There are three possible pathways for the purpose of treatment: (1) RNAi-targeted genes inhibit neuronal differentiation of stem cells; (2) the targeted genes of RNAi regulate the migration of stem cells toward the injured sites; (3) the RNAi transfers from the stem cells to other cells directly or indirectly, leading to inhibition of targeted genes.

Based on the findings summarized above, we propose that stem cells can be used as vectors for transferring RNAi-mediated genetic materials and that RNAi-modified stem cells have potential for treating CNS diseases through various pathways after transplantation (Figure 1). There are at least three different pathways involved in these processes. (1) The RNAi-targeted genes are those that inhibit differentiation of stem cells into neurons. Silencing these genes via RNAi triggers intracellular signaling to direct neural differentiation and increase the number of neurons, i.e., neuronal regeneration which compensates neuron loss following neurological disorders; (2) The targeted genes of RNAi are involved in the migration of stem cells toward the injured sites. Combination of the RNAi with stem cells increases residing of stem cells in injured tissues in traumatic diseases such as spinal cord injury and stroke; (3) The RNAi transfers from stem cells to other cells directly or indirectly, where the RNAi subsequently inhibits the expression of targeted genes. The direct way is through cell–cell contact, while the indirect way includes the release of RNAi from the stem cells and the uptake by other cells.

There are multiple pathways affecting the combination of RNAi and stem cells therapies. A better understanding of stem cell biology is needed to help discover different relative genes and develop targeted gene therapies. It has been noted that RNAi can sometimes produce nonspecific, off-target effects, leading to unpredicted phenotypes; this is attributed to miRNA-like binding in the 32 UTRs [20]. For instance, shRNA-mediated doublecortin knockdown produces untoward neuronal migration, which is associated with the disruption of endogenous let7 miRNA or Dicer [126]. Therefore, it is necessary to develop novel, safe, and effective RNAi delivery approaches. Transplantation of RNAi-modified stem cells may represent a promising treatment for neurological disorders in the future.
